# Supplementary material for: Preoperative CT anthropometric measurements and pancreatic pathology increase risk for postoperative pancreatic fistula in patients following pancreaticoduodenectomy
Source: PLoS One. 2020 Dec 3;15(12):e0243515. doi: 10.1371/journal.pone.0243515 (PMC7714124; doi:10.1371/journal.pone.0243515)
Supplement: S1 Table — (DOCX) [file pone.0243515.s002.docx]

**S2 Table. Comparison of clinical characteristics in subjects with and without visceral obesity**

|  | **Visceral obesity (-)**  **(n=37)** | **Visceral obesity (+)**  **(n=70)** | **p-value** |
| --- | --- | --- | --- |
| **Age, years** | 62.7±11.27 | 67.5±.8.86 | 0.028 * |
| **Male, no.** | 15 (40.5) | 49 (70) | 0.003 * |
| **Body mass index, kg/m^2^** | 21.31±2.87 | 24.26±2.46 | <0.001 * |
| **Skeletal muscle index, cm^2^** | 109.56±28.87 | 130.73±27.74 | <0.001 * |
| **Abdominal circumference, cm** | 76.65±7.55 | 87.85±6.73 | <0.001 * |
| **Presence of sarcopenia, no.** | 22 (59.5) | 38 (54.3) | 0.608 |
| **Soft pancreatic texture, no.** | 22 (59.5) | 40 (57.1) | 0.817 |
| **Operation time, minutes** | 424.46±78.99 | 459.14±65.36 | 0.017 * |
| **Transfusion, no.** | 18 (48.6) | 25 (35.7) | 0.194 |

Note. Data are presented as mean ± standard deviation, or number of subjects with percentage in parentheses. * are the parameters with p<0.05. Visceral obesity; visceral fat area≥100cm^2^
