# Supplementary material for: Preoperative CT anthropometric measurements and pancreatic pathology increase risk for postoperative pancreatic fistula in patients following pancreaticoduodenectomy
Source: PLoS One. 2020 Dec 3;15(12):e0243515. doi: 10.1371/journal.pone.0243515 (PMC7714124; doi:10.1371/journal.pone.0243515)
Supplement: Attachment — Submitted filename: Responses to Reviewers.docx [file pone.0243515.s003.docx]

**Responses to Reviewers’ Comments**

Manuscript ID: PONE-D-20-24280R1

Manuscript title: Predictive value of preoperative CT for postoperative pancreatic fistula in patients following pancreaticoduodenectomy

**Reviewer #3.**

1. I agree with reviewer 1, that points out that pancreatic texture hardness/softness is a subjective measure, though from clinical experience I agree that a 'soft' pancreas is more likely to lead to a leak. I accept that the authors have responded to the reviewer's comments: I would add that perhaps the authors add a sentence or two in the limitations of their work that this is a subjective measure, and more work needs to be done to be able to characterise pancreatic tissue density (and also reference the work relating to MR elastogram etc) within the actual paper.

Thank you for your advice. We added that the subjective nature of the surgeon's perception of the pancreas’ texture was a potential limitation in this study and that further research is warranted to quantitatively characterize the pancreatic texture using methods such as MR elastography or US elastography.

2. Can the authors just clarify which multivariate logistic regression was used, it is mentioned it is stepwise, but was it forwards or backwards entered?

We used the backward stepwise method for multivariable logistic regression analysis. This has been included in the statistical analysis portion of the text.

3. I may have missed it in the text, but re: the CT performed preoperatively, I note that the median time was 14 days, but why was it done? i.e. as part of the preoperative staging? Secondly re: CT postoperatively, I note that the exclusion criteria was any CTs beyond 50 days were excluded, but again in your unit did all your patients have CT scans post operatively, or was it due to post operative management looking for a complication?

The CT scans were performed as a preoperative assessment in all subjects. Regarding the exclusion criteria, the “interval between CT and surgery >50 days” was deemed to be misleading. The interval was corrected to 40 days in the previous revision. Therefore, we worded the sentence as “interval between preoperative CT and surgery >40 days” in the text. All subjects routinely underwent postoperative CT scanning for postoperative complications.

4. I think the title is slightly misleading, as the title suggests that the CT scans preoperatively can identify potential patients who develop postoperative pancreatic fistula - however even in the abstract the finding of patients with pathologies other Pancreatic adenocarcinoma or pancreatitis is a significant finding (but not a radiological diagnosis!). Hence I would probably alter the title to reflect factors that may lead to POPF instead??

Thank you for the suggestion. Accordingly, we changed the title to "Preoperative CT anthropometric measurements and pancreatic pathology increase risk for postoperative pancreatic fistula in patients following pancreaticoduodenectomy" to reflect factors that could cause POPF.

**Reviewer #4**

1. Authors should analysis the previous established risk factor of CR-POPF such as drain amylase fluid on postoperative day 1 if they use final diagnosis of the tumor.

According to the 2016 update issued by the International Study Group of Pancreatic Fistula, POPF can be diagnosed when any measurable volume of drain fluid on or after postoperative day (POD) 3 with amylase levels exceeding 3 times the upper limit of normal amylase for each specific institution is detected. Although several studies have been conducted to understand the impact of day 1 drain amylase in predicting POPF after pancreaticoduodenectomy, they all presented different cutoff values for day 1 drain amylase concentration [1]. Molinari et al. [2] reported that a drain amylase value >5000 U/L was a predictive factor for pancreatic fistula development. Jin et al. [3] suggested an amylase level of 2365 U/L in the drainage fluid as the optimal cutoff value for predicting pancreatic fistula.

Drain amylase fluid levels on POD 1 may indeed be a useful marker for the identification of POPF. However, further evaluation is needed to determine the optimal cutoff value. Moreover, since other well-established risk factors for POPF, such as sex, body mass index (BMI), pancreatic duct size, pancreatic texture, blood transfusion, intraoperative blood loss, and operation time, were included in our study, we believe the result to be meaningful.

2. Authors should show the clinical characteristics of patients who with visceral obesity and those without.

Thank you for your advice. We have added a supplementary table containing the clinical characteristics of the subjects with and without visceral obesity.

**S2 Table. Comparison of clinical characteristics in subjects with and without visceral obesity**

|  | **Visceral obesity (-)**  **(n=37)** | **Visceral obesity (+)**  **(n=70)** | **p-value** |
| --- | --- | --- | --- |
| **Age, years** | 62.7±11.27 | 67.5±.8.86 | 0.028 * |
| **Male, no.** | 15 (40.5) | 49 (70) | 0.003 * |
| **Body mass index, kg/m^2^** | 21.31±2.87 | 24.26±2.46 | <0.001 * |
| **Skeletal muscle index, cm^2^** | 109.56±28.87 | 130.73±27.74 | <0.001 * |
| **Abdominal circumference, cm** | 76.65±7.55 | 87.85±6.73 | <0.001 * |
| **Presence of sarcopenia, no.** | 22 (59.5) | 38 (54.3) | 0.608 |
| **Soft pancreatic texture, no.** | 22 (59.5) | 40 (57.1) | 0.817 |
| **Operation time, minutes** | 424.46±78.99 | 459.14±65.36 | 0.017 * |
| **Transfusion, no.** | 18 (48.6) | 25 (35.7) | 0.194 |

Note. Data are presented as mean ± standard deviation, or number of subjects with percentage in parentheses. * are the parameters with p<0.05. Visceral obesity; visceral fat area≥100cm^2^

3. In discussion (Page 11, Line 18), author speculated that the difficulty of operation in patients with greater visceral fat is the reason of high rate of CR-POPF. Do authors have objective data that shows the difficulty of pancreas anastomosis.

Obesity has negative effects on the surgical outcome following pancreaticoduodenectomy according to other studies [4–5]. The technical difficulty of treating obese patients may contribute to the development of POPF. Our study also showed that the operation time was significantly longer in subjects with visceral obesity than in those without. Future research focusing on the effect of visceral obesity on pancreatic fistula after pancreatic resection is needed.

4. Moreover, do authors have objective data that excessive visceral fat is associated with insulin resistance and other morbidities in their study.

Thank you for your comment. There is no objective data on this topic in our research because our study focused primarily on the risk factors for POPF. However, many scientific review papers have assessed the role of visceral obesity as an emerging risk factor for type 2 diabetes, atherosclerosis, and cardiovascular disease. More research is needed on the mechanism by which visceral obesity causes POPF. However, we have incorporated additional references into the text.

5. In discussion (Page 12, Line 18), authors stated “The mechanism by…soft pancreas parenchyma”. Is it correct? Many studies reported that PDAC decreased the risk of POPF.

Thank you for your observation. This was a mistake, and we have replaced it with “pathologies other than PDAC or chronic pancreatitis.”

6. Please describe about drain management in Method. Did authors place drain in all patients? And, did authors measure amylase level of drain fluid in all patients?

At our institution, external drains were inserted in proximity to the pancreatic anastomosis during each surgery, at which time the amylase level of drain fluid was routinely measured. We have added this information to the manuscript.

7. In statistics, authors used P-values of <0.05 on univariate analyses to select factors that were entered into multivariate analysis. This cut off of p-value is not common so that author should explain why they used this method.

Multivariate analysis using all variables are ideal because the statistical program automatically applies and analyzes the significant variables, but its suitability depends on the situation. When many explanatory variables are present, only variables with a p-value of 0.05 or less (i.e., statistically significant variables) as determined by univariate analysis could be included in the multivariate analysis. Researchers can perform statistical analysis in several different ways; however, as pointed out by the reviewer, we re-analyzed the results. When multivariate analysis was performed on all variables, the following results were obtained, and visceral fat and pathology were still significant variables.

| **Variable** | **Odds ratio** | **95% CI** | ***p*-value** |
| --- | --- | --- | --- |
| Sex [female] | 23.401 | 3.03, 180.280 | 0.002* |
| Skeletal muscle | 0.948 | 0.130, 0.984 | 0.005* |
| Visceral fat | 1.015 | 1.004, 1.026 | 0.007* |
| Pathology [PDAC or pancreatitis] | 29.753 | 2.691, 328.934 | 0.006* |

Note. Reference categories are in square brackets. CI: confidence interval, VF/SMI: visceral fat to skeletal muscle index ratio. * represents the parameters with p <0.05.

**References**

1. Liu Y, Li Y, Wang L, Peng CJ. Predictive value of drain pancreatic amylase concentration for postoperative pancreatic fistula on postoperative day 1 after pancreatic resection: An updated meta-analysis. Medicine (Baltimore). 2018;97(38):e12487. <https://doi.org/10.1097/MD.0000000000012487> PMID: 30235751

2. Molinari E, Bassi C, Salvia R, Butturini G, Crippa S, Talamini G, et al. Amylase value in drains after pancreatic resection as predictive factor of postoperative pancreatic fistula - Results of a prospective study in 137 patients. Annals of Surgery. 2007;246(2):281-287. <https://doi.org/10.1097/SLA.0b013e3180caa42f> PMID: 17667507

3. Jin S, Shi XJ, Wang SY, Zhang P, Lv GY, Du XH, et al. Drainage fluid and serum amylase levels accurately predict development of postoperative pancreatic fistula. World J Gastroenterol. 2017;23(34):6357-6364. <https://doi.org/10.3748/wjg.v23.i34.6357> PMID: 28974903

4. Park CM, Park JS, Cho ES, Kim JK, Yu JS, Yoon DS. The effect of visceral fat mass on pancreatic fistula after pancreaticoduodenectomy. J Invest Surg. 2012;25(3):169-173. <https://doi.org/10.3109/08941939.2011.616255> PMID: 22583013

5. Shamali A, Shelat V, Jaber B, Wardak A, Ahmed M, Fontana M, et al. Impact of obesity on short and long term results following a pancreatico-duodenectomy. Int J Surg. 2017;42:191-196. <https://doi.org/10.1016/j.ijsu.2017.04.058> PMID: 28461146
